# Fluid modernities: the birthing pool in late twentieth-century Britain

**DOI:** 10.1136/medhum-2023-012820

**Published:** 2024-06-26

**Authors:** Victoria Bates, Jennifer Crane, Maria Fannin

**Affiliations:** 1 History, University of Bristol, Bristol, UK; 2 Geographical Sciences, University of Bristol, Bristol, UK

**Keywords:** reproductive medicine, midwifery, pregnancy, History, obstetrics

## Abstract

Birthing pools are a common feature of maternity units across Europe and North America, and in home birth practice. Despite their prevalence and popularity, these blue or white, often bulky plastic objects have received minimal empirical or theoretical analysis. This article attends to the emergence, design and meaning of such birthing pools, with a focus on the UK in the 1980s and 1990s. Across spheres of media, political and everyday debate, the pools characterise the paradoxes of ‘modern maternity’: they are ‘fluidly’ timeless and new, natural and medical, homely and unusual, safe and risky. Beyond exploring the contradictions of ‘modern maternity’, we also make two key interventions. First, we contend that modern maternity has substantially expanded in recent decades to hold and include additional ideas about comfort and experience. Second, we flag the culturally specific notions of ‘modernity’ at play in modern births: the popularity of the birthing pool was typically among white, middle-class women. We argue that birthing pools have had an impact at a critical moment in birthing people’s care, and we map out the uneven and unjust terrains through which they have assumed cultural and medical prominence.

The birthing pool is today found in many maternity units in Europe and North America but is underexamined as a feature of modern birth. Such pools—used in homes as well as hospitals—are indeed often considered a particularly novel feature of late twentieth-century childbirth, but at the same time waterbirths are described as timeless, natural and commonplace ‘throughout history’ (Balaskas and Brainin 1993, 23). Birthing pools, then, have long been something of a paradox that characterises elements of late twentieth-century childbirth: both timeless and modern, natural and medical, homely and unfamiliar. The importance of the birthing pool for scholars of ‘modern’ childbirth lies in these very contradictions. This article provides the first scholarly examination of the birthing pool to show how multiple—seemingly contradictory—features of modernity could be held at once, through the history of one object. In doing so, it provides both a detailed history of an underexamined object, drawing on a range of recent interventions in this area, and also makes a broader point about the complexities of ‘modern’ maternity (Millar Fisher and Winick 2021; Parrish Morgan 2023; Weingarten 2023).

The birth pool is, at first glance, perhaps not ‘modern’ at all. Its emphasis was on embodied, personal experience rather than on ‘rationality’, ‘progress’ or ‘science’. It embraced ideas of the ‘natural’ and was envisioned as a counter-balance to high-technology medicine. However, this article argues that—on closer examination—the birthing pool actually expanded, rather than rejected, older models of modern medicine grounded in hygiene and risk management. Although the birthing pool was branded and advocated for as an object of ‘care’ and experience, in practice its introduction into homes and hospitals triggered a number of debates grounded in older ideas about ‘modern’ medicine, such as a concern with sterility and safety. It would be a mistake, then, to claim that the birthing pool (or what it represented in experience-centred maternity care), replaced older models of ‘modern’ healthcare. Instead, it expanded the idea of what modern maternity care was. The birthing pool also shows that this expanded vision of modern maternity was not neat or easy in the 1980s and 1990s. The inclusion of ‘experience’ as part of good healthcare needed to be negotiated and navigated, and there was a constant balancing act between concerns over safety and the desire to make experience central to birth. Interrogating the birthing pool through this lens shows that some of the ideas traditionally associated with modernity in medical history—such as the rejection of tradition or the idea of ‘backwardness’—were not always applicable, and that modern medicine could embrace the ‘natural’ as part of the modern. This aligns with some of the arguments that scholars of hospital history have made in other contexts (see Adams 1999).

This case study also raises bigger questions about what ‘modernity’ itself means, both as a category of analysis and in the late twentieth century. In general terms, ‘modernity’ describes *both* ‘major social and material changes… [and] the growing consciousness of the novelty of these changes’ (Gilbert, Matless, and Short 2003, 2). Yet, beyond this, the ‘periodisations, geographies, characteristics and promise’ of ‘modernity’ are, Miles Ogborn has written, ‘elusive’ and ill-defined (Ogborn 1998, 2). There are always multiple modernities that coexist at any given time, and modernity itself is often an unstable category (Chatterjee 1997; Waddington 2021). Due to the breadth and ubiquity of this concept, scholars have long noted that it may lose value as an analytical framework (Hugon 2009). This article playfully deploys the framework of ‘fluid modernity’, drawing on Zygmunt Bauman’s insights that ‘liquid’ is ‘snapshot’, ‘constantly ready (and prone) to change’ (Bauman 2000, 2). We argue that the capaciousness of the concept ‘modernity’ does not render it redundant. Rather, it uniquely enables it to capture the multiple meanings held by a birth pool, and the contradictions of ‘modern maternity’, in the late twentieth century.

Birthing pools were both a reaction against modernity and a way of redefining it. ‘Modernity’ in the context of birth was very specific and did not necessarily fit the bigger cultural models of the ‘modern’ that historians often associate with twentieth-century healthcare. This specificity is important to recognise, as modern maternity—as shown through the birthing pool—was able to hold together a number of ostensibly contradictory characteristics, such as tradition and innovation, experience and safety, nature and science, and more. It was also, in many ways, an aspirational form of ‘modernity’ that was gendered and shaped by class, race and location and was not in practice accessible to everyone. Finally, this multilayered form of modern modernity was unstable and constantly being discussed or renegotiated: pregnancy and birth were, as Tania McIntosh states, ‘at the same time intensely private and uniquely public’ (McIntosh 2012, 2).

The rise of the birthing pool is an international story, but this article focuses on the UK in the 1980s and 1990s as a case study. This article will first discuss the emergence and appearance of the birthing pool. Second, the article discusses the birthing pool as ‘experience-centred’ and ‘aspirational’. Third, the article explores the design of birthing pools, and their critical situation within movements to make hospitals ‘homely’ spaces, as well as interest in making them comfortable or ergonomic for birthing people. Finally, the article explores the pressures on birthing pools to fit within a ‘safe’ and ‘sterile’ hospital environment: these concerns did not override growing cultural and on-the-ground interest in having a ‘homely’ waterbirth, but were layered on top of it, constructing these increasingly contested objects. Overall, then, as Michelle Millar Fisher and Amber Winick write, ‘the arc of human reproduction is full of incidents in which designers imposed their values, beliefs, and convictions onto their viewers, users, and consumers in the name of finding solutions or solving problems ‘for them,’ many times without informed – or any – consent’ (Millar Fisher and Winick 2021, 22). Examining, historicising and theorising the birthing pool offers a contribution to challenging and understanding key negotiations that have shaped birthing people’s lives.

## The emergence of birthing pools

The birthing pool has a long and global history. While sadly this has not yet been written, contemporary accounts often flag, briefly, such disparate histories of waterbirth as written about in 1805, then ‘rarely broached’ thereafter (Odent 1983, 1476), or as related to ‘legends of South Pacific islanders giving birth in shallow sea water and of Egyptian pharaohs born in water’ (Thomas n.d). Supporters of this method often pay most attention to the significant role of ‘dissident childbirth assistant’ Igor Charkovsky in bringing this idea to Russia in the 1980s (Belooussova 2002, 12). A scholarly account of ‘natural childbirth’ in Russia, written by Ekaterina Belooussova, suggests that the first four water deliveries took place in spring 1980 in Moscow as an experiment, and that this practice extended, gradually, in particular through waterbirths conducted by ‘successors’ trained by Charkovsky himself (Belooussova 2002, 12). Ideas of waterbirth emerged in France in the same decade, under the influence of Dr Michel Odent. Odent had trained broadly across various fields of surgery in the 1950s (Odent 1999) and then developed birthing rooms—one with a pool—at his small state hospital in Pithiviers, 85 km south of Paris. Here, by 1983, he completed his 100th birth under water, in their pool—‘installed close to the homely birthing room’ (Odent 1983, 1476).

This narrative has become a cultural script, used to justify the use of birthing pools through reference to various geographies and histories. Beyond famous ‘pioneers’, though, interest in waterbirth grew across Europe and North America in the 1980s. In Britain, throughout this decade, birthing in water gained more media attention and, according to advocates, became more popular. Growing interest drew on and extended the ‘natural childbirth’ movement, which had taken off in the 1950s and continued in the 1980s. Key proponents in that movement, such as Sheila Kitzinger, collected materials on waterbirths within personal archives (see Wellcome Collection n.d). The birth pool, though, represents more than debates around ‘natural’ birth alone. Indeed, its development, in the 1980s and 1990s, also drew centrally on later ideas of patient ‘experience’ and ‘choice’ in healthcare, and this is visible in key Department of Health publications in the early 1990s (Hanson 2004; Raphael 2010; Bourke 2021). By 1992, recommendations from health policymakers were that all UK maternity units ‘make full provision whenever possible for women to choose the position which they prefer for labour and birth with the option of a birthing pool where this is practicable’ (Department of Health 1992, xcviii).1 Similar recommendations were made by the Royal College of Midwives and the United Kingdom Central Council for Nursing, Midwifery and Health in 1994.

It is difficult to know whether birthing pools really were as widely embraced as the traditional narrative—of their rise as a ‘global phenomenon’ from the 1980s onwards—indicates (Balaskas and Brainin 1993). Statistics on their use in the UK in the 1980s and 1990s seem to vary, raising questions about their reliability. In 1993, *The Daily Telegraph,* citing a Royal College of Obstetricians and Gynaecologists study, stated that only 1 in 10,000 births were in water, and that around 70 had taken place in 1990 (Pallot 1993, 1). Other estimates of their use range from 80 hospitals with birthing pools in 1993 to a much more extensive use of water for labour and birth in bathtubs or purpose-built birthing pools in all 219 National Health Service (NHS) provider units (where each unit could include between 1 and 8 maternity hospitals) surveyed for a Department of Health study of waterbirth in England and Wales (see Balaskas and Brainin 1993; Alderdice *et al* 1995). Beyond hospitals, there were also hundreds of portable pools for hire to use in homes and hospitals. Overall, despite these varied estimates, it seems clear that the numbers of births that included water at some point have increased steadily and significantly since the 1990s: by 2019, a Care Quality Commission report suggested that 11% of birthing people who delivered vaginally did so in water, and that this rate had increased since 2007 (Aughey *et al* 2021, 2). Yet, while increasingly in use, and of much cultural interest and recognition, the use of these pools was still, as one Mass Observer stated in 1993, often seen as somehow ‘really way-out’ and unusual (Mass Observation Project 1993, T2543).

In recent years, most people who requested birthing pools have apparently used them for pain relief but still ultimately gave birth out of the water, and the majority still opted for so-called ‘land births’ throughout the 1980s and 1990s. As discussed further below, the birthing pool was more popular among certain demographic groups and—although brought into being as an aspirational part of the ‘birth experience’ in the final decades of the twentieth century—it was often expensive to hire for individuals, or difficult to access in hospitals. Newspapers shared stories from distraught women who had aimed for waterbirths, in theory, but arrived at hospital with cervical dilation too far along or with too complex a case for this to be realised (*The Times* 1992, 12). There is a question, then, about the somewhat outsized influence of birthing pools in the public imagination and varyingly as a distinct symbol of ‘modern’, ‘natural’ or holistic maternity care, when in practice their use remained relatively rare due to both choice and availability. The birthing pool was ‘evocative and emotionally charged’, rather than one of the less visible birth objects recently charted in *Designing Motherhood*, and it was, as *Stroller* argues for the pram, both metaphor and object (Millar Fisher and Winick 2021, 18; Parrish Morgan 2023, 4, 115). Taking the birthing pool as a starting point, we may understand modern maternity anew.

## Birthing pools and the aspirational ‘birth experience’

In the UK, growing interest in birthing pools came at—and arguably as part of—a very specific time of transformation in maternity care. Hospital-based childbirth had grown dramatically under the new National Health Service since its creation in 1948, from 40% of births in 1937 to 65% by 1957 (Ministry of Health 1959). This was initially in part about demand, but also, from 1970, key policy reports recommended hospital birth as the ‘safe’ option (DHSS 1970). The shift to hospital-based birth opened up a number of conversations in society, politics, healthcare and activism about modern childbirth. Notably, the idea that hospital birth was necessarily ‘safer’ was critiqued by contemporary researchers (Campbell *et al* 1984; Tew 1990). Over the course of the 1980s, there was a shift in the tone of these conversations, particularly under the influence of feminist and birth activist groups. In particular, there was a growing emphasis on the ‘birth experience’ and a general agreement that safety should not be the only way of defining the success of maternity services (Bates, Crane, and Fannin Forthcoming). Scholars of childbirth and reproduction have written hugely useful accounts of maternity in this period, often analysing the uneasy and ever-shifting balance between arguments about ‘natural’ and ‘medicalised’ childbirth (Hanson 2004; Raphael 2010). By 1998, it is worth noting for context that 99% of births took place ‘in some kind of institutional setting, usually an NHS hospital with full obstetric facilities’ (McIntosh 2012, 1).

Joanna Bourke has written that, in the mid-twentieth century, the term ‘natural birth’ ‘could mean almost anything’ (Bourke 2021, 99). In part as a result of this, the birthing pool became entwined with the broad ideas of ‘natural birth’, and with ideas of ‘birth experience’, care and scientific endeavour. This multitude of ‘fluid’ visions is visible even in the first accounts of waterbirth. Odent, for example, framed this type of birth in a multitude of ways—as ‘natural’, ‘homely’ and enabling women to be ‘primal’, and wrote that many patients felt ‘an irresistible attraction to water’ (Odent 1983, 1476). The comparison with animals and idea of the ‘primal’ was also significant for Igor Charkovsky. Elena Tonetti-Vladimirova, who worked with Charkovsky in 1982, later wrote that he believed that birth in water would ‘relieve a baby’s brain from the shock of gravity’ and noted that ‘whales and dolphins have a much better use of their brains’, because of their smooth entry into the world (Tonetti 1995).

While on the one hand describing nature and the primal, Odent simultaneously wrote that many benefits of waterbirth were attributable to science—this was a clinical technology, as it enabled ‘the reduction of the secretion of noradrenaline and other catecholamines; the reduction of sensory stimulation when the ears are under water; the reduction of the effects of gravity’ (Odent 1983, 1476). Other benefits, however, were ‘difficult to rationalise’: these were transcendent, ethereal and sensory (Odent 1983, 1476). Odent found ‘that the mere sight of the water and the sound of it filling the pool are sometimes sufficient stimuli to release inhibitions so that a birth may occur before the pool is full’ (Odent 1983, 1476). Water ‘seems to help many parturients reach a certain state of consciousness where they become indifferent to what is going on around them’; it ‘seems to help women lose their inhibitions’, and many vocalised this, ‘cry[ing] out freely during the last contractions’ (Odent 1983, 1476). The birth pool was thus a fluid object from the outset. This multitude of theorisations would continue to feature in discussions between and among midwives, obstetricians and natural birth advocates in subsequent years.

Such a myriad of benefits were indeed reiterated and repeated by advocates who had given birth at Pithiviers. In *Birth* journal (aimed at medical audiences), for example, in 1983, a pictorial essay from a mother and childbirth educator, Peggy Quinlan, praised the birth experience of this space. Quinlan stated that key forces in her decision to give birth here were the lack of pain relief, the ‘home-like atmosphere’ and the ‘possibility of relaxing in the heated pool during labor’ (Quinlan 1983, 187). Echoing Odent on the primal benefits of such a birth, Quinlan wrote that the water ‘allows the labor to be coordinated by the primitive brain, which governs physiological processes’ (Quinlan 1983, 187). Quinlan was able to use the pool during her labour and explained that it was of sensory benefit also: ‘[t]he floating sensation was wonderful’. Additionally, she felt that the use of the pool also eased her birth: ‘[w]ith each contraction I could clearly visualize the cervix opening and stretching around the baby’s head’ (Quinlan 1983, 190).

The National Childbirth Trust (NCT) was increasingly influential in advocating for ‘natural’ methods of childbirth in hospitals, including labouring and delivering in water, from the 1970s and 1980s. This sometimes meant giving birth with as little medical intervention as possible. Natural birth in the UK context did not necessarily imply giving birth outside of the hospital; it could just mean embracing more natural forms of pain relief, though it did often also involve an emphasis on midwife-attended home births and midwife-led hospital birth (including, since the 2000s, a rise in midwife-led birth centres) (Hanson 2004; Raphael 2010). NCT magazines for members, such as *New Generation*, echoed the ideas of Odent around waterbirth as particularly primal, for example discussing the ‘natural attraction to water in labour’ and the idea that women are ‘Aquatic animals’ (Balaskas 1988, 5). This organisation also emphasised that people should be offered autonomy and choice during pregnancy and birth. To quote one member, who hired a pool ‘from a couple in Birmingham’ in 1989 for her second birth, ‘Michel Odent has a birthing pool in the labour room at his French clinic, so shouldn’t we have a few in Shropshire?’ (Williams 1989, 11)

NCT members writing in the late 1980s thus positioned waterbirths as contentious but also thought they should be offered them. The birthing pool was pitched as a ‘natural’ object here, rather than explicitly as a ‘modern’ or high-technology one; at the same time, ‘modern’ childbirth was partly defined by the power and autonomy to choose a ‘natural’ birth. This emphasis on the power to choose was part of another trend in modern healthcare at this time: the patient as ‘consumer’ (see Mold 2011; O’Hara 2013). Education and health systems across Europe, in a similar period, were positioning families—rather than individuals—as active ‘agents’ or partners in the delivery of the welfare state (Crane 2023). Though much of the British literature emphasised woman-centred maternity care at this time, differing slightly from the language of family-centred birth evident in other contexts such as North America (Enkin 1984; Fannin 2003), it is significant that one of the letters cited here was from a ‘couple’; the birthing pool was seen as part of a family experience. Without ignoring the important feminist roots of ideas about female choice and women-centred maternity (see Creed and Marland 2023), the two trends were very similar in practice: modern childbirth meant giving families control over their environment, whether that meant having a partner present for birth or choosing to have a birthing pool. The idea that choice was central to birth would gain momentum in subsequent years, culminating in the *Changing Childbirth* report of 1993 (McIntosh 2012, 134–44).

Ideas of desiring not only a safe birth, but also a ‘birth experience’ reached new heights in the 1980s and 1990s (Bates, Crane, and Fannin Forthcoming). In this context, proponents presented the waterbirth as especially aspirational. Shared accounts of ‘experience’ disseminated by mainstream newspapers and activist publications emphasised that such births were particularly ‘mystical’ and ‘extraordinary’ (MacDonald 1993, 5)—the atmosphere would be ‘magical, very hushed and peaceful’ (Melaniphy 1989, 11). Typically, however, these benefits accrued within a hospital setting, rather than in a space that fundamentally disrupted the norms of modern maternity: one gushing article in the *Daily Telegraph* from 1993, for example, stated that a father had described waterbirth as ‘a joyful and extraordinary experience – providing it is carried out in hospital with proper medical supervision’ (MacDonald 1993, 5). Notably, this birth method was not ‘aspirational’ because it would alleviate pain: an NCT article warned against this assumption. Rather, it was the transcendence of this experience—the primal elements described above—that made such a birth desirable. *The Times* (1993) newspaper published an editorial against this in 1993, arguing that the birthing pool symbolised a broader cultural expectation that women should feel ‘guilty’ about opting for pain relief, instead, ‘obliged by fashion or peer pressure to reject it’ (*The Times* 1993).

Campaigners and advocates of waterbirth sought to present this ‘aspiration’ as commonplace and universal. NCT materials from the 1980s and 1990s, for example, stated that, ‘[w]hen word of successful home water birth began to circulate, women began to clamour for this facility to be available in hospitals’ (Corbishley 1989, 10). Yet, at the same time, the cost of waterbirths was hugely prohibitive, and that these objects were sold and distributed via a market meant they were by no means universally available, but rather managed and distributed dependent on knowledge, time, resources, geography and cash. Newspaper reports refer to the cost of hiring specific pools for waterbirths as £150 per month in 1991, plus £25 for the necessary disposable lining (Byrne 1991, 17). For women who did obtain them, that cost shaped their birth experience: one NCT member told *New Generation* that, ‘as we had paid the cost of hiring the tub, I wanted a good long soak to get my money’s worth’ (Melaniphy 1989, 11). Other birthing people, of course, did not have this option: one respondent to a Mass Observation directive about birth, published in 1993, wrote that, ‘I wanted a water birth but found that having a pool was expensive’ (Mass Observation Project 1993, B2031).

This specific form of ‘aspirational’ birth was not appealing to, or appropriate for, everybody. In response to the 1993 Mass Observation Directive about birth experience, one respondent discussed how they had given birth and then immediately given up their child for adoption. They wrote that:

Obviously I wish things could have been different. I wish I had a husband I loved, one or preferably two children in the family. I would like to have tried one of the natural birth methods, but not a water birth because I do not feel that is natural for human beings. (Mass Observation Project 1993, 2175)

This rich source gives a brief glimpse into a sense that the idea of a waterbirth was caught up in a broader cultural script about the ‘aspirational life’ in the 1990s: what women should aim and yearn for, and the lives they should lead. The birthing pool was immediately tied to ideas that women should aim to have a husband and one or two children, yet also, still, seen as a slightly unusual part of that formulation: potentially not ‘natural’ and in some way controversial. Yet—in part due to paucity of evidence, in part due to the power of the personal experience in this area—campaigners around the birthing pool never directly addressed the fact that this object may be a preference, in particular for white, affluent women. They lobbied and demanded the expansion of this technology, without directly analysing who this spending would benefit, and who it would further obscure; or indeed how a birthing pool, if indeed it brought about pain relief for all, could be designed and constructed as equally accessible to all.

Race, indeed, must not be neglected in the narrative of the birthing pool as aspirational. Our accounts about birthing pools from the 1980s and 1990s do not attend to the significance of race as a category, yet this fundamentally shapes the contexts of modern maternity. Innovative projects such as the Young Historians Project, conducting oral histories with elders of African and Caribbean heritage, have made more visible the critical work of Black nurses in midwifery and maternity, in the mid-to-late twentieth century (Young Historians Project 2022). Despite this, patient experience remains hugely differentiated by race. As a 2019 report in the UK (and parallel report in the USA) flagged, Black women in the UK have been five times more likely to die in childbirth than white women and Asian women four times more likely to die; Black women in the USA have been four times more likely to die than white women (with even higher disparities in certain states) (MBRRACE-UK 2019). Research for campaigning organisation Five X More, surveying 1300 Black and Black mixed women in 2022, highlighted issues with the attitudes, knowledge and assumptions of healthcare professionals (Five X More 2022). Recent books, such as *Designing Motherhood*, seek to analyse the ‘racist, misogynistic motivations’ frequently ‘woven’ into objects of maternity, yet also recognise that narratives from minoritised women are too rarely reflected in existing ‘vehicles and platforms that promulgate the histories of *design’* (Millar Fisher and Winick 2021, 21).

In this context, then, we must be cautious about the idea that it was aspirational to move beyond questions of ‘risk’ into experience-centred design. For many people, particularly Black and Asian women, childbirth was an endeavour in which there were increasing calls for attention to safety, rather than a focus on features such as birthing pools. Recent reports—such as by Five X More— call for change in the attitudes of healthcare practitioners, rather than specific additions such as a pool. This is not to assume, of course, that the birthing pool was not an aspiration at all for Black women. Private centres in America, with Black obstetrician-gynaecologists at the forefront, primarily serve people of colour and do offer such facilities (About the Birth Centre of New Jersey! 2023). However, it is important to think also about the gaps in our archival records; as Five X More notes, ‘Black women’s voices and lived experiences have been notably absent from the literature’ on childbirth until recently (Five X More 2022, 7). It is crucial to recognise that a focus on ‘experience’ and the call for a demedicalisation of the birth environment required great confidence in the safety of hospital-based birth, which not every person felt.

Much of the literature that we have on UK birthing pools in the late twentieth century, which informs our claims that they were aspirational, is dominated by white middle-class voices. It is very difficult to know whether birthing pools were equally aspirational among other groups, for whom safety might have been a greater concern. Recent studies of waterbirth use have also suggested that cultural factors might play a role: ‘[w]omen from ethnic minority (particularly Asian) communities were perceived by midwives as being less likely to use a pool. Midwives [in the UK] suggested this may be due to unfamiliarity with waterbirth, reluctance to remove clothing, or the views of relatives attending the birth’ (Milosevic *et al* 2020, 4). Though historical studies of waterbirth use do not tend to offer demographic detail, a study from 2015 to 2016 showed that waterbirth was less common in women under 25, obese women, Black and Asian women, and women from less affluent areas (Aughey *et al* 2021). To return to a point made earlier, there have always been multiple modernities, and the birthing pool was perhaps only part of the good ‘modern’ birth for some people. The birthing pool had multiple fluid allures for some, but these did not universally resonate.

## Designing birthing pools

The birthing pool was constructed as part of a shift towards holistic and person-centred modern healthcare, which constructed an abstract ‘patient’ or ‘service user’ without reference to race, ethnicity, disability or class. For those who *did* have the privilege to aspire and afford to use birthing pools, they carried a particular social and cultural symbolism. In addition to representing choice, control and a more natural model of birth, they apparently helped to make hospital spaces more inviting and comfortable through their design. When situated in place, the birthing pool facilitated the bringing together of ‘modernity’ and ‘homeliness’ in interior design and could apparently facilitate more personalised and inclusive spaces. There was not necessarily always a tension between the ‘modern’ and the ‘antimodern’ or traditional, or rather this tension was primarily a rhetorical one. The birthing pool shows how modern medicine was being redefined to (re)*incorporate* traditional values, rather than in juxtaposition with them.

**Figure 1:**
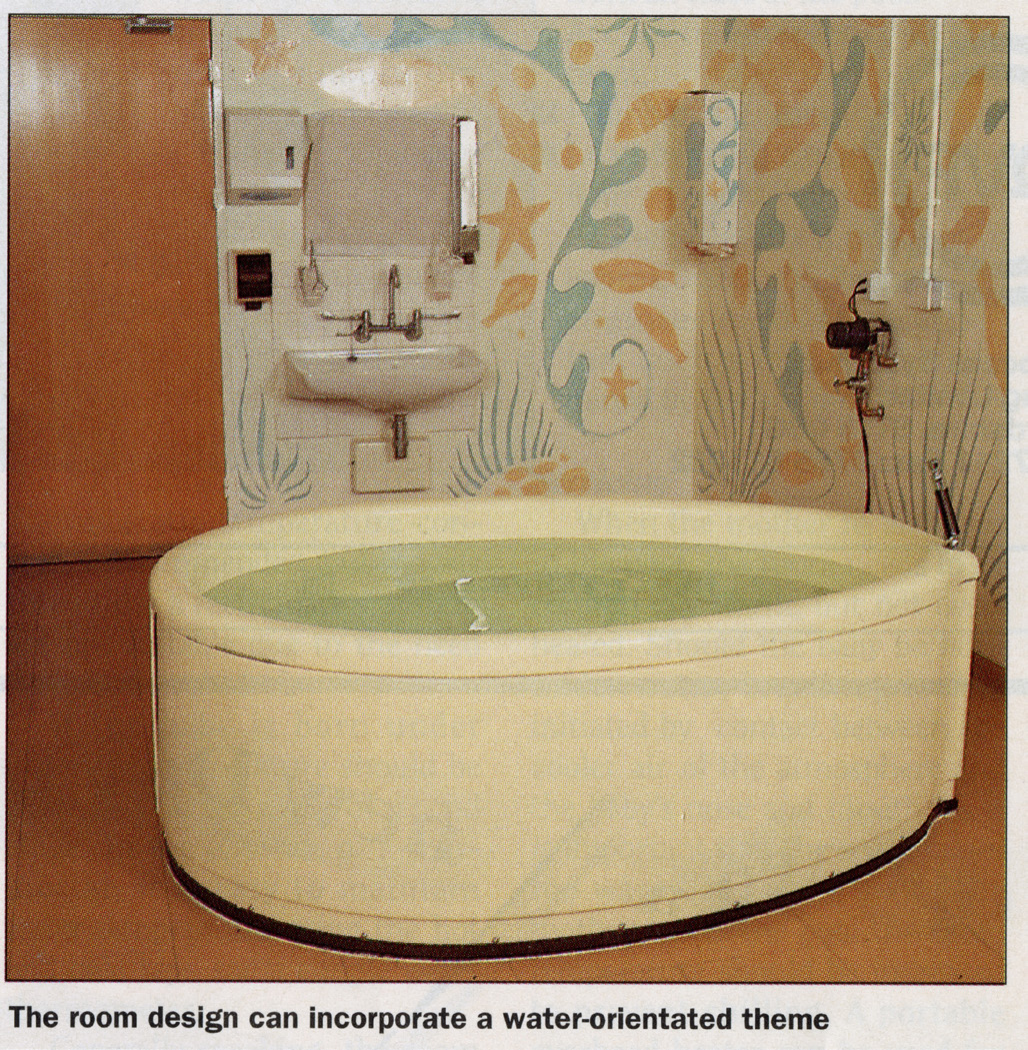
Reproduced from Balaskas and Brainin (1993) 23, with thanks to Keith Brainin for permission to publish. Image from *Hospital Development* courtesy of RIBA Collections.

At first, birthing pools took a range of forms. In the absence of consistent support or provision from the NHS in this area, some women and midwives got creative and used various vessels, fashioning birthing pools for use at home. An article in *New Generation* discussed an independent midwife in Brighton who had delivered 13 babies in water, using a mini-skip (Corbishley 1989, 10). Other sources discussed birth in jacuzzis, hired pools and ‘a plastic fish tank’ (Williams 1989, 11). In hospitals, though, the birthing pool was increasingly part of a more considered design agenda and was often introduced in dialogue with architecture and interior design. In some hospitals, this process was framed as part of making a more ‘homely’ atmosphere. This ‘homeliness’ was never meant to emulate the home precisely, but rather to evoke the feelings associated with the home such as safety, security and comfort (Duque *et al* 2019; Richards and McLaughlan 2023). A ‘home-like environment’ was also increasingly conflated with patient-centred care by the 1990s (Shearer and Gray 1994, 15). Birthing pool design, and the relationship of birthing pools to hospital architecture and design, played an important part in such efforts to demedicalise maternity environments. Keith Brainin and Janet Balaskas, who sold birthing pools and were an important part of the early birthing pool movement, emphasised that they were part of making an ‘intimate and homely’ atmosphere and should be situated in appropriate interior design including ‘warm tones … for wallpaper, curtains, blinds and cushions, to give the feeling of a bedroom’ (Balaskas and Brainin 1993). This suggestion, then, meant that hospital birth would better emulate a home birth. They also suggested a ‘water-oriented theme’ (Balaskas and Brainin 1993). Figure 1 shows the example that they shared in their article in *Hospital Development*: though it is at first glance a somewhat sterile, round, white birthing pool, it also emulates a ‘homely’ bathroom when combined with a water-oriented theme.

Balaskas was, for Tania McIntosh, influential, alongside Sheila Kitzinger, in creating a ‘new battleground’ of birth, ‘between women and everything that represented control and power, whether it was doctors or midwives, machinery or protocols’ (McIntosh 2012, 10). Balaskas’ work in this area, then, and her attempts to use birth pools to make hospital birth ‘homely’ and ‘comforting’, must be read within a broader picture of reshaping institutional landscapes away from perceived obstetric ‘control’.

Indeed, birthing pools existed in relation to numerous human and non-human elements in making ‘homeliness’ and other atmospheric conditions. In 1983, for example, the Maternity Services Advisory Committee noted that:

While staff attitudes more than anything create a friendly and welcoming atmosphere, a reception area and delivery rooms which look as unclinical and homely as possible will help to achieve this aim. Much can be done at comparatively low cost by the use of wallpapers, curtains and other soft furnishings. (Maternity Services Advisory Committee 1983)

**Figure 2:**
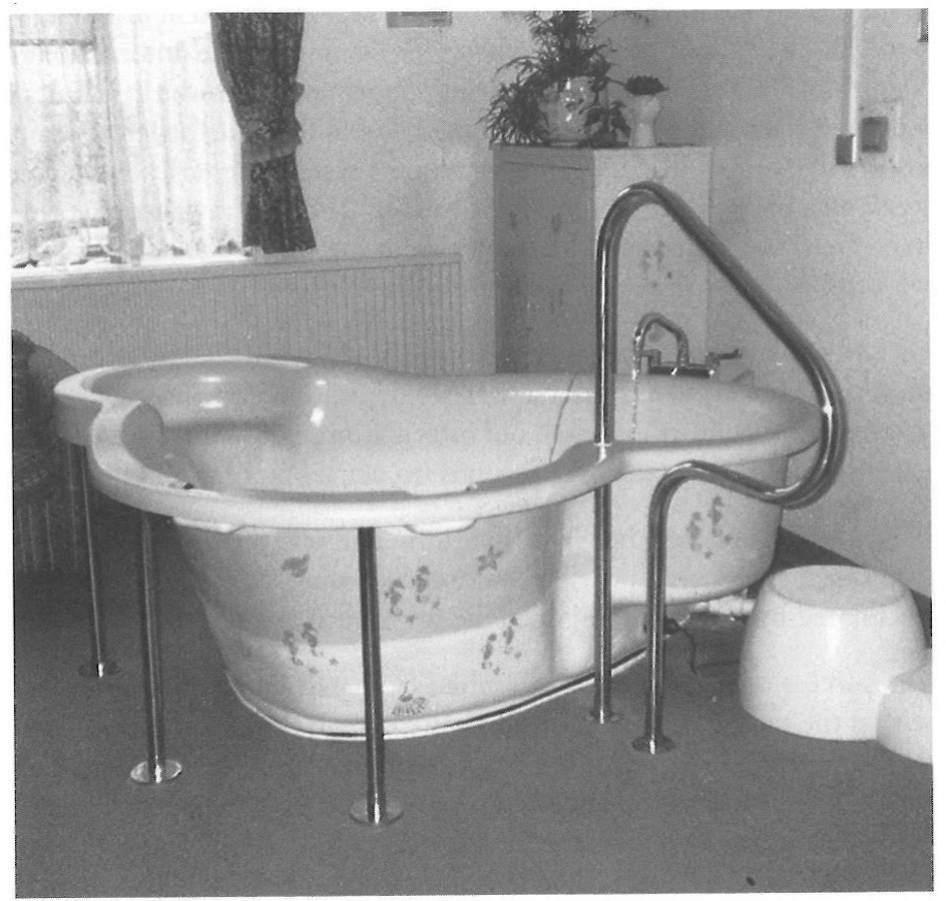
Image of a Northampton Birthing Pool from the mid-1990s. Reproduced from Garland (2000), 139. Permission granted.

Birthing pools were part of this model of homeliness, both in the recommendations for interior design to accompany them, and as part of a ‘friendly and welcoming atmosphere’. Similar themes come through in memories of people’s use of birthing pools, in which the pool itself is part of a welcoming atmosphere: to quote one NCT member, ‘[a]As I entered the labour room, all I saw was the bright blueness of the birthing tub and the smiling faces of midwife Diena and Sue’ (Melaniphy 1989, 11). The birthing pool in this memory combined with the ‘attitudes of staff’ to create a ‘friendly and welcoming atmosphere’. From our own informal observations of birthing spaces, it is clear that some staff—particularly in midwife-led areas—also make birthing spaces ‘homely’, by adding decorative features such as colourful stickers (see also Duque *et al* 2019). Though evidence of these kind of interventions is difficult to find historically, some pictures show subtle informal additions to spaces; figure 2, for example, shows seahorse stickers and house plants in a birthing space that were presumably added in a somewhat ad hoc way. However, birthing pools were unusual in being a *single* object that could be a marker of homeliness, care and all the other complex features of modernity discussed here, such as cleanliness and technology.

As ‘homeliness’ became entrenched in the hospital by the 1980s, the hospital was also moving more and more into the home. This might be seen, in part, as a way to embrace the whole family in the birthing process. As a ‘low’ technology, homely object, birthing pools also included features such as plumbing. When they were brought into homes, they therefore brought a technology *in* and changed the nature of the home environment. One Mass Observer in 1993 described their hired ‘large fiberglass fish pond aprox [sic] 8 ft X 6ft X Ap[p]rox 2 ½ ft it was really well designed with layers I could sit on, got foam to lay on the layers and bottom and a hose incoming from hot/cold tap and a hose going out with a pumb rigged up to a drill to pumb [sic] water out to put more hot in’ (Mass Observation Project 1993, B2031). The plumbing needed to ensure a constant temperature was a frequent feature of these accounts but did not seemingly undermine the status of the birthing pool as a ‘homely’ or ‘natural’ item. It was able to hold these different versions of modern maternity together. In this sense, the birthing pool is perhaps most closely aligned with another complex medical (low) technology, the hospital bed. This was an object that similarly combined ideas of care, technology, and even homeliness (see Arnold-Forster and Bates 2024; Willis, Goad, and Logan 2018). The birthing pool—in theory, at least—added new layers and levels of complexity to this version of homely modernity. It brought in the element of choice over the environment, as well as more ‘natural’ elements such as water, and round shapes.

Birthing pools were important sites of negotiation in relation to questions of choice and control. This meant not only being able to choose a birthing pool in the first place—although with all the limits of access noted above, in practice—but also being able to control and shape the experience of being *in* the birthing pool. This personalisation agenda was part of a wider trend in hospital design, and particularly maternity design. The 1983 Maternity Services Advisory Committee recommendations noted that, ‘[i]f possible, the delivery rooms and sitting areas should have windows, and artificial lighting should be adjustable in intensity and focus. The temperature and humidity in each room should be adjustable to suit the needs of mothers and their babies’ (Maternity Services Advisory Committee 1983). The emphasis on making spaces ‘adjustable’ was particularly important in childbirth spaces, as part of the feminist renegotiation of authority and power in birthing. Birthing pools allowed for personalisation in terms of choosing to have a waterbirth, and in the context of home births people could potentially choose from a wide range of pool designs. There were, though, limits to the degree to which people were allowed to control their birthing pool environments. Midwives typically still controlled the water to remain at ‘body temperature’, an idea that linked to some of Odent’s ideas about natural birth and water, but over time there was a growing argument that pool users should be able to choose the temperature for their own comfort (see Royal College of Obstetricians and Gynaecologists/Royal College of Midwives 2006).

Part of the apparent value of birthing pools was that they were inclusive, particularly of different body types. In theory, they allowed for a birth that was personalised in the sense of providing a more comfortable option for people who struggled with traditional birthing furniture or beds, though there is a weight limit. In practice, the ergonomics of birthing pools needed refinement, and they were not as inclusive as they first appeared. As with other important hospital furnishings such as the hospital bed, it was difficult to balance the needs of hospital staff with the comfort of the patient. New designs were developed to make them more accessible and easier for midwives to use. In the meantime, hospital designers and staff often made their own adaptations, such as adding handrails and steps, or using birthing balls for midwives to sit on while accompanying someone in the pool (Chartered Institute of Ergonomics and Human Factors 2020; Health and Safety Executive 2018). In many ways, then, the claim that birthing pools offered choice, control, personalisation and inclusivity was an ideal rather than a reality. Early birthing pools were often difficult to use, expensive to hire and largely controlled by midwives. However, it remains important that there were ongoing debates about these issues and adaptations to design and use. Birthing pools did not just articulate new ideas about modern maternity but were sites in which the meanings—and materialities—of modern maternity were negotiated.

## Birthing pools as safe and sterile

Birthing pools were introduced into hospitals in a mode of experimentation. Even as they became more common in hospitals in the late 1980s, there was no professional consensus regarding their safety and effectiveness. They were never introduced as a straightforward object of holistic ‘care’ alone, despite many policy-makers’ and medical practitioners’ efforts to carve them out as objects of experience rather than medical technologies. They were always hybrid objects, of care and healthcare, of experience and safety, of nature and technology and of choice and control. Whatever the discursive efforts were to brand the birthing pool as one thing or the other, in practice the material object itself always held—in its design and its use—all of these features of modern maternity. There was a constant negotiation between them, for example between experience and safety, and thus the maternity pool also held within it the challenges and tensions of modern childbirth. Its introduction into hospitals drew attention to the question of balancing experience-centred childbirth with rigorous attention to risk, cleanliness and safety.

As the birthing pool became increasingly prevalent, and entered hospital spaces, concerns around safety and sterility reshaped debate. Advocates of birthing pools had long cited health benefits alongside more experience-based ones, and questions about evidence and safety soon followed. To quote one article in *Hospital Development* magazine by Brainin and Balaskas from 1993, birthing pools not only apparently improved comfort but also offered medical benefits such as ‘fewer second degree perineal tears’ (Balaskas and Brainin 1993, 23). There were extensive debates about whether such benefits were proven, though campaigners made statements such as, ‘[t]here have been no randomised controlled trials of water birth, but a reservoir of experience is building up’ (Corbishley 1989, 10). Over time, as birthing pools became more established as part of NHS infrastructure and ‘DIY’ pools became less common, such questions around safety became increasingly important to both the design and use of the pools. In this sense, then, it would be a mistake to present the birthing pool too neatly as an object of care or experience, or even as straightforward evidence of a model of childbirth that is less concerned with risk. In practice, the birthing pool was increasingly shaped by concerns about safety and risk management over time and became the site for negotiating the tensions between these different aspects of modern modernity in the late 1980s and 1990s.

Early proponents of the pool—such as Odent—had sold the ‘natural’ benefits of the birthing pool, positioning it explicitly as not requiring disinfection. In his original article on the birthing pool published in 1983 in the *Lancet*, Odent wrote: ‘[t]he water is ordinary mains tap water, at a temperature of 37°C. The water is not sterilised and contains no chemicals or additives of any sort’ (Odent 1983, 1476). Yet, the idea of the water as ‘natural’ or non-sterilised quickly dissipated among even early sellers of birthing pools. See, for example, a description in the *Guardian* from 1991 of an 'average' pool available to hire:

It is 25.5 inches deep, to cover the breasts when sitting down, and is made of either a non-porous plastic or acrylic, both with a non-slip surface and resistant to bacteria. The lining is thick blue PVC and the design conforms to hygiene regulations, with separate disposable pipes for incoming and outgoing water. (Byrne 1991, 17)

This description, and others from sale catalogues, design magazines and newspapers, recognises the white or blue nature of the pool; although they were sometimes represented as ‘homely’, these colours were also associated with sterility and cleanliness (Bates 2023). The nature of the materials of these pools—‘non-porous’ and ‘resistant to bacteria’—was also framed around cleanability, and, as in the *Guardian* article, descriptors often wished to confirm that a pool ‘conforms to hygiene regulations’ (Byrne 1991, 17). Balaskas and Brainin explained that such materials were carefully chosen: alternatives such as ‘GRP fibre glass’ were ‘generally not recommended as they are more porous and prone to leeching and osmosis’ (Balaskas and Brainin 1993, 25)

Those designing and constructing birthing pools in the UK, then, thought hard about how to make these pools easy to clean, looking to ensure both that birthing people felt comfortable and safe within them, and also that they could be sold to hospitals. Yet, these manufacturers nonetheless reported struggling to bring their pools into hospital spaces. Those looking to sell birthing pools had to consider plumbing, size, the structural integrity of floors to support this weighty object, heat, and ventilation. This brought them into contact with a range of professionals. As *New Generation* magazine wrote, describing a conference around waterbirth held in Maidstone Hospital in 1989, ‘[o]bstetricians, electricians, structural engineers and works managers were all very obstructive, and needed a lot of convincing… Infection control nurses were horrified’ (Corbishley 1989, 10). Describing a different barrier, the following year, the *Observer* reported that Brainin encountered the ‘usual objections’ of hospitals to birthing pools, in particular that ‘we don’t have room to set it up’ as ‘[m]ost hospitals are built like fortresses’, yet the author also noted that ‘without a structural survey we can’t disprove this objection’. Looking to forestall this, Brainin’s next pool was designed to sit where hospital baths had formerly sat, and to be of similar size and reliant on the same plumbing (Cooper 1990, 49). Such obstacles were likewise recalled by Beverley Beech, of the Association for Improvements in Maternity Services, recounting in 2000 former campaigning in this area. Lobbying at the same time as Brainin, she had been frustrated by—in her words—the way that ‘having inveigled women into hospital with siren calls of beautiful pools many of the staff developed tactics for ensuring that few women ever used them’ (Beech 2000, 1)[Bibr R83].

Midwives’ views of birthing pools were mixed. Although they may have been enthusiastically welcomed by some midwives early on, especially independent midwives attending home births, in professional settings midwives ‘often voice concerns over legal and professional accountability surrounding water births’ (Garland 1996, 71). Proceedings from the First International Water Birth Conference held in 1995 in London include contributions from midwives, all supportive of waterbirth, but who note the ‘huge and powerful onslaught from various obstetricians…and even some midwives’ of criticism of waterbirth on the grounds of safety (Gordon 1996, 144). Attending labour and birth in a pool presented practical challenges that required additional training and the development of skills, despite the paucity of written guidance available (Garland 2000). Assessing the progress of labour, listening to the fetal heartbeat, judging the amount of blood loss and many other tasks midwives carry out could prove much more challenging during a waterbirth. One midwife recounted that ‘labouring in water also gives women more privacy because the midwife does not have access to the woman unless the woman specifically gives the midwife that access…and the midwife is in a more subservient position than she is normally’, especially as many midwives tended not to get into the water (Flint 1996, 62). For Rosemary Jenkins (1996, 53), writing as a former practising midwife and later employed by the Royal College of Midwives, supporting waterbirth was not, for her, a matter of being a ‘passionate advocate’ but rather because of her conviction that ‘women should have more choice about their care when having a baby and anything that might enable a wider range of choice should be seriously considered’. Jenkins positions waterbirth, interestingly, as a ‘health technology’ in need of serious, evidence-led assessment ‘to be considered along with all other health technologies, to be subjected to economic analysis and clinical trial before it becomes widely disseminated in the health care system’ (Jenkins 1996, 58). Midwives too sought to navigate broader tensions between claims that waterbirth was more ‘natural’ (and therefore required fewer interventions) and ensuring that their practice is ‘evidence-based’ (Feeley 2018).

Despite the attempts of manufacturers to adapt the birthing pool, concerns about safety escalated in the early 1990s, disseminated both by concerned newspapers and professional societies. In 1993, the *Daily Telegraph* reported ‘[h]ealth chief alerted after babies die during water births’ (Pallot 1993). This ‘fashion’, the paper described, that had developed in the mid-1980s, had meant that ‘several’ babies in the UK, France and Sweden had drowned: in France, an involved couple had been charged with involuntary manslaughter. The paper reported that Professor Geoffrey Chamberlain, of St George’s Hospital in London, understood that the Royal College of Obstetricians and Gynaecologists had alerted the Chief Medical Officer, Kenneth Calman, ‘to their fears’. The article represented the issues of sterility and safety in parallel: the pools were both linked to deaths *and*, Chamberlain stated, ‘the water becomes pretty mucky and that can lead to infection’ (Pallot 1993, 15).

Issues of sterility were also explored in professional journals. A series of reflections in 1994 on infection and the use of birthing pools in *The Lancet* noted that protocols for infection control ‘seemed not to exist’ and that ‘data on available on the microbiological and virological safety of this mode of delivery were unhelpful’ (Ridgway and Tedder 1996, 1051). This led clinical microbiologists at a hospital in London to call for hepatitis (B and C) and HIV testing for prospective mothers wishing to use a birthing pool, as ‘amniotic fluid, blood, and faeces makes contamination of the birthing pool surfaces with large quantities of maternal bacteria and viruses inevitable’ (Ridgway and Tedder 1996, 1051). This policy was, the authors noted, subject to intense criticism from other medical professionals, the media and ‘midwives, patients and pressure groups’ (Ridgway and Tedder 1996, 1052). The lack of consensus about the safety or risk posed by birthing pools suggests that at this early stage of their introduction into hospitals, birthing pools crystallised ongoing debates about the role of mandatory blood tests for pregnant women in light of health policies, articulated in *Changing Childbirth*, that promoted choice during pregnancy and birth (see Brocklehurst, Garcia, and Lumley 1996; Roome and Spencer 1996).

Concerns around sterility and safety thus pushed against the simple incorporation of the birthing pool, as a technology, into home births and hospital births alike. Yet it’s important to recognise that this was not a simple story of campaigners and manufacturers wishing to introduce birthing pools, and obstetricians and hospital infection control teams resisting them. Rather, opposition and support were multilayered, complex and unpredictable. In 1993, the maternity hospital in Bristol shared information about ‘adverse outcomes’ with other hospitals and authorities after one baby died and another suffered possible brain damage, as their mothers had labours involving the hospital’s birthing pool (Marks 1993). Following this, nonetheless, the hospital’s head of obstetrics and gynaecology emphasised that these could well be ‘chance events’, and that, ‘[i]f the pool is used properly and strict criteria are in use, there should not be a significant risk’ (Marks 1993). In response, medics on this site did not remove pools, but rather lowered their temperature ‘by 5 or 6 degrees centigrade to 35 degrees during labour’, started to take hourly temperature checks of those in the pool and informed birthing people about adverse events (Marks 1993; *Reuters* 1993). For many medics involved, more broadly, birthing pools were seemingly brought into hospitals as part of thinking about experience and care, rather than being seen as a sterile, or ‘medical’ device. A United Kingdom Central Council for Nursing, Midwifery and Health Visiting (UKCC) statement on waterbirth, from 1994, stated that:

The Council recognise that waterbirth is preferred by some women as their chosen method for delivery of their babies. Waterbirth should, therefore, be viewed as an alternative method of care and management in labour and as one which must, therefore, fall within the duty of care and normal sphere of the practice of a midwife. Waterbirth is not considered to be a ‘treatment’. (UKCC 1994 cited in Beech 2000)

The emphasis on care rather than treatment or therapy signals how the birthing pool was introduced to hospital settings as a technology of experience as well as a potentially effective way to reduce obstetric interventions. At the same time, by the early 2000s, an obstetrician speaking to a House of Commons inquiry would question whether waterbirth was, itself, now a ‘form of intervention’ in maternity (House of Commons Health Committee 2003, 34). This shows the power of the space of a hospital to reshape objects within it: once accepted by the majority of NHS hospitals, this object was, by at least one obstetrician, now itself ‘an intervention’, subsumed under the landscape and possibilities of hospital birth.

While many medics supported the use of birthing pools, a story of opposition and expertise in maternity is also disrupted by the fact that many women also did not want them. One person commenting to Mass Observation, without having given birth themselves, stated, ‘[i]t looked rather dangerous to me. There is some controversy about this method’ (Mass Observation Project 1993, L2307). Angela Davis has argued, through powerful oral history research, that women who had children after 1970 often rejected home birth, considering it ‘old-fashioned’, ‘the preserve of eccentrics’ or ‘something that was never even an option’ (Davis 2012, 96–97). Again, while this is absent from material that has been stored and archived, it is worth acknowledging that, in ‘modern’ maternity of recent years, Black women face huge and disproportionate dangers in hospital births. Likely, in the past, the birthing pool, hired by an individual and used as part of a home birth, could represent a form of safety, avoiding the danger associated with clinical spaces—but we also have too few sources to explore this. For research to interrogate the breadth of ‘modern maternity’, beyond the experiences of highly vocal white advocates, we must be open to seeing the birthing pool—and other such objects—as safe and dangerous, sterile or dirty, across and between groups.

## Conclusion

The importance of the birthing pool for scholars of modern childbirth lies in its contradictions. As an object, it holds together many of the complexities of modern healthcare and more specifically of modern birth, where modern birth involves tensions, borne often by birthing people themselves, between safety and risk, choice and aspiration, and the investments of birth as an ‘experience’. The birthing pool shows that, in practice, modern childbirth brought together many apparently oppositional concepts. The birthing pool was simultaneously a sterile medical device and reproductive technology, and a ‘homely’ object of experience and care. By the 1980s and 1990s, ideas of ‘modern’ childbirth were ‘fluid’ and incorporated all these ideas. The birthing pool is one of many objects that hold together these different features of modernity, particularly in hospital settings. However, it is also unique in the way it brought together specific issues, for example questions around what it meant to have a ‘natural’ birth in a hospital setting. Some interventions in hospitals were clearly designed to improve experience—for example decoration, sensory design—while others focused on safety or pain relief. The birthing pool was unusual in the ways that it held these different ideas together at once.

Focusing on the 1980s and 1990s in the UK, this article has shown that ideas about experience-centred maternity care incorporated—rather than replaced—more risk-centred models of childbirth that dominated in the postwar period. Inclusive, comfortable, colourful childbirth spaces in which people felt in control were new features of modernity, rather than being in opposition to ‘modern’ healthcare. In short, the history of the birthing pool shows how ideas about ‘modern’ healthcare itself expanded, to hold and include additional ideas about comfort and experience. Yet, it also shows how this idea of ‘modernity’ was culturally specific and not always successful in practice; for example, early birthing pools were often expensive, or had to be adapted to be inclusive of different body types. The design of the birthing pool, then, had lived and embodied impact, but rarely attended to the ‘marginalised or unheard’ (Chidi 2021, 9). The balance between the different features of ‘modernity’, for example comfort and safety, is sometimes uneasy and often being negotiated.

In making these conclusions, it is important to understand that birth experiences can be lengthy, involving a pool but also many other forms of reproductive technology, and birth expectations and plans can differ from birth realities. Most significantly of all, the ways in which births are subsequently documented and recorded privilege certain voices and memories. Authors from medical professions have documented the majority of births—as required by health systems and governments—while only a small minority of birthing people provide written descriptions of their births. Published accounts of birth, whether from newspapers, books, memoirs or through campaign groups, have become more common over time but are still disproportionately provided by white, privileged women. Researching this article, we frequently found testimonies from the same campaigners and activists in this area, typically white women who favoured waterbirth, or their white male partners, who had enjoyed the experience or who, in some cases, were hoping to sell their own birthing pools. We have sought to recognise that birthing people’s experiences were starting to be heard in the late twentieth century, in a powerful feminist moment, but also to reflect on which women’s voices were obscured and diminished in a narrative that assumed the safety of hospital spaces. There is ample space for significant further research in this area, as well as for further investigations of the specific roles of midwives in this process, and the international connections at play in the rise of birth pools.

The birthing pool, overall, shows that ‘modern’ maternity was a complex, fluid and somewhat unstable concept that held within it several ostensible contradictions. As Erica Chidi powerfully writes, preceding the *Designing Motherhood* collection, this design can be rethought to better ‘enhance, simplify, soften, and excite whatever it touches’ (Chidi 2021, 9). Attending to the history of the birthing pool shows that its power, and the evidence for its use, emanates from a relatively small, focused group of passionate campaigners and advocates, huge believers in ‘natural birth’ and with minimal attention to the disparities of birth experience across race, ethnicity, class and disability. The birthing pool has a history of contradiction, as safe and dangerous, homely and medical—it is not necessarily an object that primarily exists as an aspiration and metaphor for the ‘good birth’ of white, privileged, women, but without input on its design, and on the broader contexts and structures of maternity provision across groups, it may become so.

## Data Availability

No data are available. All underlying data are available from sources referenced throughout this paper.

## Notes

1. When we use the term ‘woman’, this is to echo the language of the sources and texts we are evaluating. We would also like to signal our awareness that birth is experienced not only by those who identify as ‘women’ but also, for example, by non-binary people and transgender men – though these groups have often been excluded from historic debates in this area.

## References

[R1] “About the Birth Centre of New Jersey!” 2023. Available from: http://birthcenternj.com/about-us/

[R5] Adams A . 1999. “Modernism and Medicine: The Hospitals of Stevens and Lee, 1916-1932.” Journal of the Society of Architectural Historians 58 (1): 42–61. 10.2307/991436

[R6] Alderdice F. , Renfrew M. , Marchant S. , Ashurst H. , Hughes P. , Berridge G. , and Garcia J. . 1995. “Labour and Birth in Water in England and Wales.” BMJ (Clinical Research Ed.) 310 (6983): 837. 10.1136/bmj.310.6983.837 PMC2549219 7711622

[R13] Arnold-Forster A. , and Bates V. . 2024. “Care and Crisis: Making Beds in the National Health Service.” Journal of British Studies: 1–27. 10.1017/jbr.2023.138

[R8] Aughey H. , Jardine J. , Moitt N. , Fearon K. , Hawdon J. , Pasupathy D. , Urganci I. , Harris T. , and NMPA Project Team . 2021. “Waterbirth: A National Retrospective Cohort Study of Factors Associated with Its Use among Women in England.” BMC Pregnancy and Childbirth 21 (1): 256. 10.1186/s12884-021-03724-6 33771115 PMC8004456

[R84] Balaskas J . 1988. “Water Birth.” New Generation 5.

[R10] Balaskas J. , and Brainin K. . 1993. “Design Guide – Birthing Pool Units.” Hospital Development: 23–25

[R12] Bates V . 2023. “Cold White of Day: White, Colour, and Materiality in the Twentieth-Century British Hospital.” Twentieth Century British History 34 (1): 1–37. 10.1093/tcbh/hwac020

[R11] Bates V. , Crane J. , and Fannin M. . Forthcoming “The Construction and Politics of ‘Birth Experience’ in Britain, 1948-93.” Cultural History

[R16] Bauman Z . 2000. Liquid Modernity. Polity.

[R83] Beech B . 2000. “Waterbirth - Time to Move Forward.” AIMS Journal. Available from: https://www.aims.org.uk/journal/item/waterbirth-time-to-move-forward. Accessed 4 Sep 2023.

[R17] Belooussova E . 2002. “The ‘Natural Childbirth’ Movement in Russia: Self-Representation Strategies.” Anthropology of East Europe Review 20 (1). https://scholarworks.iu.edu/journals/index.php/aeer/article/view/381.

[R22] Bourke J . 2021. “Becoming the ‘Natural’ Mother: Power, Emotions, and ‘Natural’ Childbirth between 1947 and 1967.” Past & Present 246: 92–114. 10.1093/pastj/gtaa031

[R23] Brocklehurst P. , Garcia J. , and Lumley J. . 1996. “Birthing Pools and Infection Control.” Lancet (London, England) 348 (9022): 275. 10.1016/s0140-6736(05)65592-4 8684232

[R25] Byrne J . 1991. “Testing the Water.” Guardian: 17

[R27] Campbell R. , Davies I. M. , Macfarlane A. , and Beral V. . 1984. “Home Births in England and Wales, 1979: Perinatal Mortality According to Intended Place of Delivery.” British Medical Journal (Clinical Research Ed.) 289 (6447): 721–24. 10.1136/bmj.289.6447.721 6434055 PMC1442830

[R28] Chartered Institute of Ergonomics and Human Factors . 2020. “Case Study 18: Improving Birthing Pool Design.” Available from: http://activebirthpools.com/wp-content/uploads/2023/01/Improving-birthing-pool-design.pdf. Accessed 1 Sep 2023.

[R29] Chatterjee P . 1997. Our Modernity. Rotterdam & Dakar: Sephis.

[R87] Chidi E . 2021. “Prologue.” In Designing Motherhood: Things That Make and Break Our Births, 9. Cambridge, MA: The MIT Press.

[R30] Cooper E . 1990. “Throwing the Baby in with the Bath Water.” Observer: 49

[R19] Corbishley H . 1989. “Water Birth Workshop.” New Generation 10.

[R31] Crane J . 2023. “Agents of Change? Families, Welfare and Democracy in Mid-to-Late Twentieth Century Europe.” Contemporary European History 32 (3): e1. 10.1017/S0960777322000078 PMC7617402 39991013

[R32] Creed F. , and Marland H. . 2023. “Improving Maternity Care through Women’s Voices: The Women’s Health Strategy Continues a Long Process of Advocacy, History and Policy.” Available from: https://www.historyandpolicy.org/policy-papers/papers/improving-maternity-care-through-womens-voices-the-womens-health-strategy-continues-a-long-process-of-advocacy. Accessed 10 May 2024.

[R33] Davis A . 2012. Modern Motherhood: Women and Family in England, 1945-2000. Manchester: Manchester University Press.

[R26] Department of Health . 1992. “Changing Childbirth and the Maternity Services Report, ‘Winterton Report.’” London HMSO

[R68] Department of Health and Social Security . 1970. “Domiciliary Midwifery and Maternity Bed Needs.” London: HMSO

[R34] Duque M. , Pink S. , Sumartojo S. , and Vaughan L. . 2019. “Homeliness in Health Care: The Role of Everyday Designing.” Home Cultures 16 (3): 213–32. 10.1080/17406315.2020.1757381

[R36] Enkin M . 1984. “Family-Centred Obstetrics.” In Pregnancy Care for the 1980s, edited by Zander L. and Chamberlain G. . London: Palgrave.

[R37] Fannin M . 2003. “Domesticating Birth in the Hospital: ‘Family‐Centered’ Birth and the Emergence of ‘Homelike’ Birthing Rooms.” Antipode 35 (3): 513–35. 10.1111/1467-8330.00337

[R38] Feeley C . 2018. “12 What Evidence Informs Midwifery Clinical Practice When Women Make Birthing Decisions That Are Outside of Guidelines? – An Empirical Study of UK Midwives Working in the NHS.” BMJ Evidence-Based Medicine 23: A6–7. 10.1136/bmjebm-2018-111024.12

[R39] Five X More . 2022. “The Black Maternity Experiences Survey: A Nationwide Study of Black Women’s Experiences of Maternity Services in the United Kingdom.” Available from: https://static1.squarespace.com/static/5ee11f70fe99d54ddeb9ed4a/t/628a8756365828292ccb7712/1653245787911/The+Black+Maternity+Experience+Report.pdf

[R40] Flint C . 1996. “Water Birth and the Role of the Midwife.” In Water Birth Unplugged: Proceedings of the First International Water Birth Conference, edited by Beech B. A. L. , 60–62. Hale: Books for Midwives Press.

[R41] Garland D . 1996. “The Role of the Midwifery Supervisor.” In Water Birth Unplugged: Proceedings of the First International Water Birth Conference, edited by Beech B. A. L. , 70–76. Hale: Books for Midwives Press.

[R42] Garland D . 2000. Waterbirth: An Attitude to Care. Hale, Cheshire: Books for Midwives Press.

[R43] Gilbert D. , Matless D. , and Short B. , eds. 2003. Geographies of British Modernity: Space and Society in the Twentieth Century. Oxford: Blackwell Publishing.

[R86] Gordon Y . 1996. “Water Birth - the Safety Issues.” In Water Birth Unplugged: Proceedings of the First International Water Birth Conference, edited by Beech B. A. L. . Hale: Books for Midwives Press.

[R44] Hanson C . 2004. A Cultural History of Pregnancy: Pregnancy, Medicine and Culture 1750-2000. Basingstoke: Palgrave MacMillan.

[R45] Health and Safety Executive . 2018. “Manual Handling Risks to Midwives Associated with Birthing Pools: Literature Review and Incident Analysis.” Available from: https://www.hse.gov.uk/research/rrpdf/rr1132.pdf. Accessed 1 Sep 2023.

[R47] House of Commons Health Committee . 2003. “Choice in Maternity Services Ninth Report of Session 2002-3, Volume 1.” Available from: https://publications.parliament.uk/pa/cm200203/cmselect/cmhealth/796/796.pdf

[R48] Hugon A . 2009. “Maternity and Modernity in the Gold Coast, 1920s–1950s.” Ghana Studies 12 (1): 77–95. 10.1353/ghs.2009.0004

[R49] Jenkins R . 1996. “Assessing the Effect of a New Health Technology.” In Water Birth Unplugged: Proceedings of the First International Water Birth Conference, edited by Beech B. A. L. , 53–59. Hale: Books for Midwives Press.

[R51] MacDonald V . 1993. “A Water Birth That Went Swimmingly Well.” Daily Telegraph 5

[R52] Marks K . 1993. “Hospital Tells of Baby Death under Birth-Pool Labour.” Daily Telegraph

[R53] Mass Observation Project . 1993 “Responses to the 1993 Autumn/Winter Directive Part 2, Birth Experience.” Available from: https://www.massobservationproject.amdigital.co.uk/Documents/Detail/1993-autumnwinter-directive-part-2/9732727

[R75] Maternity Services Advisory Committee . 1983. “Redrafting of the second report 'Care During Childbirth'; report on the revision of Hospital Building Note No. 21", The National Archives, JA 382/20.

[R54] MBRRACE-UK . 2019. “Saving Lives, Improving Mothers’ Care: Lessons Learned to Inform Maternity Care from the UK and Ireland Confidential Enquiries into Maternal Deaths and Morbidity, 2015-2017.” Available from: https://www.npeu.ox.ac.uk/assets/downloads/mbrrace-uk/reports/MBRRACE-UK%20Maternal%20Report%202019%20-%20WEB%20VERSION.pdf

[R55] McIntosh T . 2012. A Social History of Maternity and Childbirth: Key Themes in Maternity Care. Milton Keynes: Routledge.

[R21] Melaniphy O . 1989. “Water Baby in a Hurry.” New Generation 11.

[R56] Millar Fisher M. , and Winick A. . 2021. Designing Motherhood: Things That Make and Break Our Births. Cambridge, MA: The MIT Press.

[R57] Milosevic S. , Channon S. , Hughes J. , Hunter B. , Nolan M. , Milton R. , and Sanders J. . 2020. “Factors Influencing Water Immersion during Labour: Qualitative Case Studies of Six Maternity Units in the United Kingdom.” BMC Pregnancy and Childbirth 20 (1): 719. 10.1186/s12884-020-03416-7 33228569 PMC7682119

[R66] Ministry of Health . 1959. “Report of the Maternity Services Committee (Cranbrook Report).” London HMSO

[R58] Mold A . 2011. “Making the Patient-Consumer in Margaret Thatcher’s Britain.” Historical Journal (Cambridge, England) 54 (2): 509–28. 10.1017/S0018246X10000646 22826610 PMC3401488

[R59] Odent M . 1983. “Birth under Water.” Lancet (London, England) 2 (8365–66): 1476–77. 10.1016/s0140-6736(83)90816-4 6140561

[R50] Odent M . 1999. “Lifeline-Michel Odent.” Lancet (London, England) 353 (9154): 764. 10.1016/S0140-6736(05)76145-6

[R61] Ogborn M . 1998. Spaces of Modernity: London’s Geographies 1680-1780. London: Guildford Press.

[R62] O’Hara G . 2013. “The Complexities of ‘Consumerism’: Choice, Collectivism and Participation within Britain’s National Health Service, c.1961-c.1979.” Social History of Medicine 26 (2): 288–304. 10.1093/shm/hks062 24771976 PMC3635502

[R63] Pallot P . 1993. “Health Chief Alerted after Babies Die during Water Births.” The Daily Telegraph: 1

[R64] Parrish Morgan A . 2023. Stroller. London: Bloomsbury.

[R65] Quinlan P . 1983. “Geneviève’s Birth at Pithiviers.” Birth (Berkeley, Calif.) 10 (3): 187–90. 10.1111/j.1523-536x.1983.tb01422.x 6557822

[R85] Raphael A.-J . 2010. “Natural Childbirth in Twentieth Century England: A History of Alternative Approaches to Birth From the 1940s to the 1990s.” Available from: https://qmro.qmul.ac.uk/xmlui/handle/123456789/1601?show=full. Accessed 5 Apr 2023.

[R3] Reuters. 15 October 1993 “Doctors Ask Britain to Investigate "Water babies””

[R69] Richards K. , and McLaughlan R. . 2023. “Beyond Homeliness: A Photo-Elicitation Study of the ‘Homely’ Design Paradigm in Care Settings.” Health & Place 79: 102973. 10.1016/j.healthplace.2023.102973 36682264

[R70] Ridgway G. L. , and Tedder R. S. . 1996. “Birthing Pools and Infection Control.” Lancet (London, England) 347 (9007): 1051–52. 10.1016/s0140-6736(96)90195-6 8606599

[R71] Roome A. P. C. H. , and Spencer R. C. . 1996. “Birthing Pools and Infection Control.” Lancet (London, England) 348 (9022): 274. 10.1016/s0140-6736(05)65590-0 8684231

[R72] Royal College of Obstetricians and Gynaecologists/Royal College of Midwives . 2006. “Joint Statement No.1.” Available from: http://activebirthpools.com/wp-content/uploads/2014/05/RCOG-waterbirth.pdf. Accessed 1 Sep 2023.

[R73] Shearer P. , and Gray J. . 1994. “Vehicle for Change.” Hospital Development: 13–15

[R74] Tew M . 1990. “Safer Childbirth?” In Safer Childbirth? A Critical History of Maternity Care. Boston, MA: Chapman and Hall. 10.1007/978-1-4899-2975-4

[R76] Thomas C . n.d. “The History of Water Birth, Baby Centre Blog.” Available from: https://www.babycentre.co.uk/a542003/the-history-of-water-birth. Accessed 19 Apr 2024.

[R2] The Times. 14 August 1992. “Birth of an Enterprise – Maternity in the Private Sector,” 12

[R4] The Times. 16 October 1993 “Unnecessary Pain – There are no medals for bravery in childbirth,” 17

[R77] Tonetti E . 1995. “What If Nothing Is Holding You Back?’, Birth into Being.” Available from: https://www.birthintobeing.com/waterbirth. Accessed 10 Jan 2024.

[R78] Waddington K . 2021. “Problems of Progress: Modernity and Writing the Social History of Medicine.” Social History of Medicine 34 (4): 1053–67. 10.1093/shm/hkaa067

[R81] Weingarten K . 2023. Pregnancy Test. London: Bloomsbury.

[R80] Wellcome Collection . n.d. “The Archive of Sheila Kitzinger (1929-2015).” Available from: https://wellcomecollection.org/works/z53cpms6

[R20] Williams L . 1989. “Naturally Relaxing.” New Generation 11.

[R79] Willis J. , Goad P. , and Logan C. . 2018. Architecture and the Modern Hospital: Nosokomeion to Hygeia. London: Routledge.

[R82] Young Historians Project . 2022. “A Hidden History: African Women and the British Health Service.” Available from: https://www.younghistoriansproject.org/_files/ugd/5865f8_7da7314224e74c77a2d98b2ea7eb9d99.pdf

